# Chromosome-Level Genome Assembly of the Meishan Pig and Insights into Its Domestication Mechanisms

**DOI:** 10.3390/ani15040603

**Published:** 2025-02-19

**Authors:** Huipeng Du, Jianchao Hu, Zhiyan Zhang, Zhongzi Wu

**Affiliations:** National Key Laboratory for Swine Genetic Improvement and Germplasm Innovation, Ministry of Science and Technology of China, Jiangxi Agricultural University, Nanchang 330029, China; dhp1561857525@outlook.com (H.D.); jianchaohu@hotmail.com (J.H.); bioducklily@hotmail.com (Z.Z.)

**Keywords:** meishan pig genome, PacBio sequencing, structural variation, domestication region, high fecundity

## Abstract

Pigs are essential agricultural animals, and among the various breeds, the Meishan pig, a native breed of China, is renowned for its high reproductive performance. This breed has been introduced to many countries to enhance local pig breeding programs. However, there have been limited genomic and population genetics studies focusing on Meishan pigs. We created a chromosomal-level genomic assembly using high-depth PacBio sequencing and Illumina sequencing data collected from a Meishan pig. Additionally, we analyzed whole-genome sequencing (WGS) data from Chinese boars and Meishan pigs to identify domestication selection signals within the Meishan breed. The assembled genome of the Meishan pig (MSjxau) was found to be 2.45 Gb in size, with a scaffold length of 139.17 Mb. The quality value was 37.06, and the BUSCO score was 96.2%, indicating good completeness, continuity, and accuracy. We annotated transposable elements, segmental duplication, and genes in the MSjxau genome. By combining these data with 28 publicly available genomes, we provide a high-quality structural variants resource for pigs. Furthermore, we identified 716 selective sweep intervals between Chinese wild pigs and Meishan pigs, where the selected gene *PGR* may be linked to the high fertility observed in Meishan pigs. Our study offers valuable genomic and variation resources for pig breeding and identifies several genes associated with the domestication of the Meishan pig. This lays the groundwork for further investigation into the genetic mechanisms behind complex traits in pigs.

## Simple Summary

This study assembled a high-quality chromosome-level genome of the Meishan pig using Illumina and PacBio sequencing technologies. By integrating this new genome with existing pig genome assemblies, we contribute to developing a pig pan-genome, facilitating research on structural variations. Selective sweep analysis between the Chinese wild boar and the Meishan pig revealed key candidate genes, such as *TBX19* and *PGR*, linked to domestication traits. These findings provide valuable genetic resources for studying Meishan pig biology and offer a foundation for improving breeding strategies and conservation efforts.

## 1. Introduction

The pig (*Sus scrofa*) is not only one of the most important farm animals in the world, but also a near-perfect biomedical model animal [1,2]. Genomics and population genetics studies are critical to understanding the genetic mechanisms of complex traits in pigs [3,4]. The advent of low-cost second-generation sequencing technology has led to a rise in large-scale whole genome sequencing (WGS) based population genetics studies in pigs, with several recent research efforts involving sample sizes of over 1000 [5,6,7]. However, in the field of swine genetics, only a limited number of causal genes or variants have been thoroughly identified, leaving the genetic mechanisms behind most phenotypic traits still largely unresolved [8,9]. One reason is that the NGS sequencing data cannot accurately determine the genotype of all genetic variants due to sequence duplication or sequencing bias, resulting in a loss of heritability [10,11]. Advances in long-read sequencing technologies and assembly algorithms have revolutionized genome assembly [12,13,14], enabling the construction of chromosome-level assemblies for multiple pig breeds [15,16,17]. Long-read sequencing platforms, such as PacBio and Oxford Nanopore, combined with Hi-C scaffolding and optical mapping, have significantly improved contiguity and completeness, leading to high-quality genome assemblies for breeds like the Bamaxiang [18], Ningxiang [19], Luchang [20], and Korean Nanchukmacdon pigs [21]. These resources have provided insights into structural variations (SVs), evolutionary history, and trait-associated genes [22,23]. To date, only 39 high-quality pig genomes have been reported in the NCBI database, representing 29 different pig breeds. The genomic resources available for pig populations are still limited in comparison to those for humans [24].

The domestication of pigs in China dates back over 9000 years, and indigenous breeds such as the Meishan (MS) pig are the most well-known domesticated pigs in the Taihu Lake region. Meishan pigs are renowned for their high reproductive efficiency, early maturity, and superior meat quality, making them one of the most important maternal lines in modern pig breeding [25]. Although a chromosome-level genome of the Meishan pig has been reported previously, this resource primarily represents individual animals [26], and little is known about the genetic structure at the population level. Additionally, while the domestication history of pigs from wild boars (*Sus scrofa*) has been studied [27,28], the genetic mechanisms that underlie the domestication of the Meishan breed are not well understood [29]. Understanding the genetic differences between Meishan pigs and Chinese wild pigs, particularly in terms of adaptation and selective breeding, is critical for both conservation and breeding efforts aimed at enhancing desirable traits [30].

Recent advances in structural variant (SV) research have highlighted the importance of population-level SV catalogs as essential resources for understanding genomic diversity and its functional implications [31]. High-quality, population-scale genome assembly data are essential for accurately detecting of large fragments and complex structural variants, which significantly contribute to the genetic variation underlying complex traits and diseases [32,33,34]. Furthermore, the algorithms like Graphtyper [35] and Paragraph [36] enable graph genome-based structural variant typing of NGS data with a sample size from hundreds to millions. Recently, a study of porcine graph-based genome analyzed 11 high-quality pig genomes [37], which may not be entirely representative of the rich genetic diversity of pigs; therefore, it is important to construct structural variant sets based on larger genomic datasets.

Here, we aim to generate high-quality chromosomal level genome assembly for Meishan pigs using a comprehensive approach combining PacBio long read sequencing and Illumina short read sequencing. The genome generated in this study is an important complement to existing pig genome resources. At the same time, we also reveal the selective domestication signal between Meishan pigs and Chinese wild boars based on the large-scale and high depth WGS resources. Our findings will contribute to a broader understanding of the genetic mechanisms of pig domestication and adaptation, with implications for breeding and conservation strategies.

## 2. Materials and Methods

### 2.1. Sample Collection

A male Meishan pig from Shanghai, China, was used for de novo whole-genome assembly. Ear tissue samples from this individual were collected, immediately frozen in liquid nitrogen, and then stored at −80 °C until DNA extraction. All animal procedures were conducted in accordance with the “Guidelines for the Care and Use of Laboratory Animals” (GB/T 27416-2014, Quality and Capability Requirements for Laboratory Animal Institutions) and were approved by the National Standards of the People’s Republic of China as well as the Ethics Committee of Jiangxi Agricultural University.

### 2.2. DNA Extraction and Sequencing of the Genome

Genomic DNA was extracted from ear tissues using Qiagen’s DNeasy Blood & Tissue Kit (Manufactured by Qiagen, Hilden, Germany). DNA concentrations were measured using Qubit 3.0 Fluorometer and Nanodrop-1000 instrument (Thermo Scientific, Waltham, MA, USA), while DNA purity and integrity were assessed by agarose (0.8%) gel electrophoresis. Paired-end genomic DNA libraries with insert sizes of 300–400 bp were constructed using the NEBNext^®^ Ultra™ DNA Library Prep Kit for Illumina (NEB, San Diego, CA, USA) according to the manufacturer’s instructions and sequenced on an Illumina Novaseq6000 platform using the paired-end 2 × 150 bp mode at Novogene Biotech, Beijing, China. FastQC [38] was used to assess the quality of the original raw data, and fastp [39] was used to remove the sequencing connectors and delete low-quality, single-ended reads to form clean Illumina data. The data sizes were 192.16 Gb, and the average sequencing depth was about 74x.

Full-length genomic DNA were extracted from ear tissue based on the standard operating manual from PacBio official. Femto Pulse System and Qubit 3 Fluorometer were used to assess the quality of DNA sequence. An above 10 kb SMRTbell CLR (continuous long reads) DNA library was constructed following the manufacturer’s protocol and then sequenced on 2 SMRT (Single Molecule Real Time) cells within a PacBio Sequel platform (Pacific Biosciences of California, Menlo Park, CA, USA) with P6/C4 chemistry at Novogene Biotech, Beijing, China. On average, this generated 23.85 million subreads, with a subread N50 of 11.73 kb. Please note that the average sequencing depth was about 87x.

### 2.3. Genome Size Estimation and De Novo Assembly

We performed quality control on Illumina data using fastp, and then used KMC (v3.2.4) [40] software to construct a k-mer frequency distribution map with k = 21. Based on the above k-mer data, we used Genomescope2 [41] software to estimate the genome size and heterozygosity of the Meishan pig.

The de novo genome assembly was performed using PacBio subreads with the Flye (v2.9.5) [42] software. The preliminary assembly was polished using the filtered Illumina paired-end reads via Pilon [43]. One round of iterative error correction was conducted to ensure the accuracy of the assembly. As a result, highly accurate contigs were identified. These high-accuracy contigs were then mounted to the chromosome level based on Sscrofa11.1 using RagTag [43], successfully constructing the chromosome-level Meishan pig genome. Subsequently, gaps in the chromosomes were filled using long PacBio reads (>10 kb) with TGS-GapCloser (v1.2.1) [44]. Finally, a final round of polishing was performed with second-generation data, completing the entire genome assembly process.

### 2.4. Genome Quality Assessment

To assess the integrity and accuracy of the newly assembled Meishan pig genome, we performed the following validation steps. First, we aligned the whole-genome sequencing short reads of the Meishan pig to the genome using BWA software(v2.2.1) [45] to estimate the accuracy of the single-base assembly. Additionally, we evaluated the quality value (QV) scores for each chromosome using yak. We also assessed the quality of the generated genome using BUSCO (v5.0.0) [46] software based on the mammalia_odb10 lineage dataset (created on 20 November 2019). Finally, we compared the synteny between MSjxau and Susscrofa11.1 using doplotly.

### 2.5. Distribution of Repetitive Elements and Genome Annotation

We used RepeatModeler (v.2.0.1) [47] to construct a de novo repeat sequence library, which was then merged with a pig-specific repeat sequence library extracted from the RepeatMasker [48] repeat database. Then, using default parameters and the constructed repeat sequence library, we annotated the repetitive elements in the assembly using RepeatMasker (v.4.0.5). The repeat landscape was visualized using tools provided by RepeatMasker, specifically the scripts calcDivergenceFromAlign.pl and createRepeatLandscape.pl. Additionally, we annotated the fragment repeats in MSjxau using seadef, filtering the results based on the following criteria: SD fragment size of at least 1 kb, and sequence similarity among two SDs greater than 90%. Furthermore, we used liftover [49] to map the gene annotation information from Susscrofa11.1 v110 to MSjxau, annotating a total of 21,042 genes.

### 2.6. Genomic Structural Variation Analysis

We downloaded nanopore/PacBio sequencing data for 11 pigs from public databases [50,51] and assembled the data into contigs using Flye [42]. Additionally, we obtained genome assembly data for 17 pigs based on third-generation sequencing data from public sources [20,26,52,53,54,55]. We aligned the MSjxau genome and the 28 aforementioned genomes to the Sscrofa11.1 reference genome, and used DipCall [56] to detect structural variations (SVs) in each genome. The structural variants from all individuals were then merged using JasmineSV [57] to form a non-redundant SV set. DipCall also reports small fragments of SNPs and indels between genome comparisons. Therefore, we used SnpEff [58] to annotate the SNPs and indels between the MSjxau and Sscrofa11.1 genomes.

### 2.7. Domestication Signal Analysis of Meishan Pigs

We downloaded whole-genome sequencing (WGS) data from 44 Meishan pigs (MS) and 45 wild boars from public databases, with sequencing depths of at least 9x, which allows for accurate identification of genetic variations. We scanned the entire genome for domestication regions using a 20 kb sliding window with a 10 kb step size. We calculated the average Fst and nucleotide diversity (Pi) within each 20 kb window using vcftools [59]. Significant differentiated regions were defined as the top 1% of Fst regions, which also had a Pi(wild boar) to Pi(Meishan pig) ratio greater than 2. GO functional enrichment analysis was performed using the R package clusterProfiler [60].

## 3. Results

### 3.1. Genome Assembly and Evaluation of the Meishan Pig

**Figure 1:**
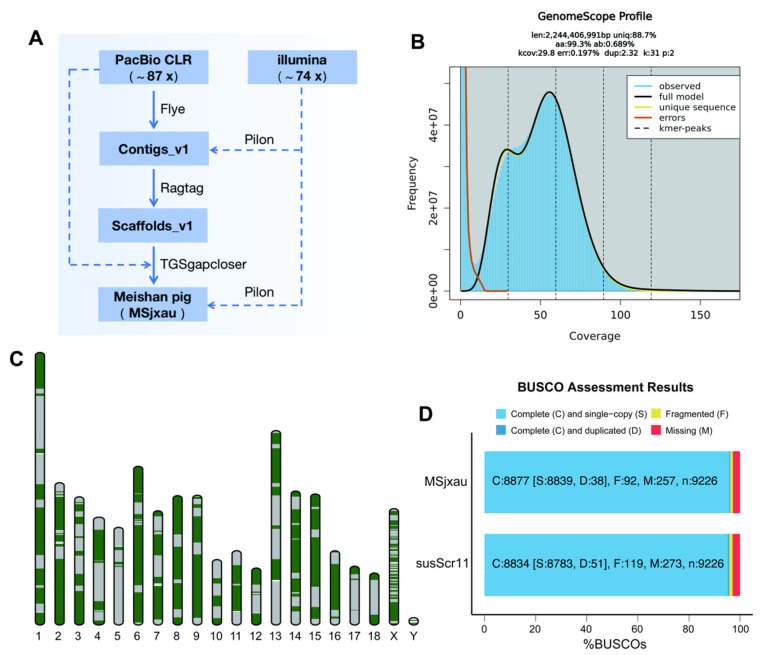
The genome assembly of the Meishan pig. (**A**) Workflow for the genome assembly of Meishan pig. (**B**) The frequency distribution of k-mer for Meishan pig genome (k = 31). (**C**) The distribution of contigs on the chromosome, with green and gray representing different contigs. (**D**) BUSCO evaluates the completeness of genome assembly.

With the assistance of high-depth PacBio CLR and NGS sequencing data, we de novo assembled the Meishan pig genome (MSjxau). The detailed genome assembly workflow is shown in Figure 1A. We generated a total of 227.88 Gb of raw PacBio data (with a read N50 of 11.73 Kb), corresponding to a sequencing depth of 87.64x, and 192.16 Gb of whole-genome NGS data, with a sequencing depth of 73.91x. We estimated the MS genome characteristics using Illumina data, and under a K-mer threshold of 31, the chromosome size was estimated to be 2.24 Gb with a heterozygosity rate of 0.689% (Figure 1B), which is close to the size of the Sscrofa11.1 genome. Using PacBio CLR reads, we assembled the MS contigs with Flye, resulting in a total length of 2,443,415,892 bp, consisting of 1921 contigs, with a contig N50 size of 15,622,572 bp. After scaffolding with Ragtag, gap filling using TGS data, and polishing with NGS data, we obtained the final MSjxau genome assembly. The MSjxau genome is 2,447,343,390 bp in size, with 98.42% of the sequences anchored to 20 chromosomes, and the scaffold N50 is 139.17 Mb (Table 1).

We conducted a comprehensive evaluation of the quality and completeness of the MSjxau genome assembly using multiple approaches. First, mapping with minimap2 revealed that 90.55% of the PacBio reads could be remapped to MSjxau, with regions supported by at least 15 PacBio reads covering 100% of the MSjxau genome. Similarly, 98.2% of the Illumina reads could be remapped to MSjxau, with regions supported by at least 15 Illumina reads covering 99% of the genome. Secondly, the Meishan pig assembly demonstrated overall high base accuracy. The base accuracy of the genome was estimated using Illumina read data, with a QV score of 37.06. The QV scores for each chromosome ranged from 30.09 to 37.80, indicating good accuracy. Furthermore, each chromosome was composed of several large contigs, suggesting good continuity (Figure 1C). Finally, BUSCO analysis of genome completeness showed that approximately 96.2% of the core conserved mammalian genes were present in the Meishan pig assembly, exceeding the 95.8% found in the Sscrofa11.1 reference genome (Figure 1D). This also indicates that the genome assembly is close to complete. These results highlight the superior quality and potential applications of the MSjxau assembly in genomic research.

### 3.2. Genome Annotation of the Meishan Pig

**Figure 2:**
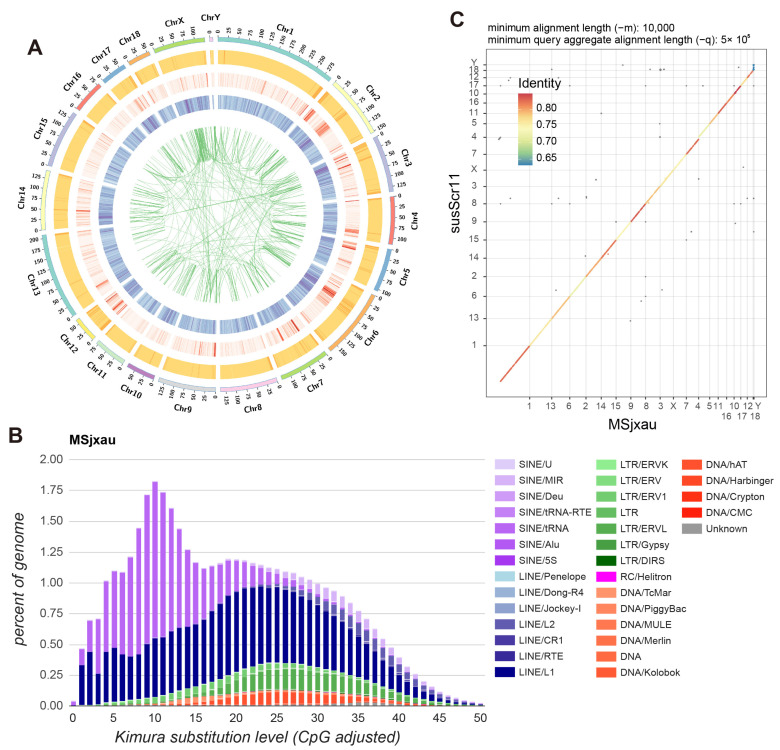
Genome annotation of Meishan pig assembly. (**A**) Distribution of features in the Meishan pig genome. For the outer to inner regions, each circle represents the GC content, transposable elements number, gene number and collinearity of repetitive sequences in the 500 kb nonoverlapping windows. (**B**) Sequence divergence of repetitive elements in the Meishan pig assembly. (**C**) Sequence consistency between Meishan pigs and Sscrofa11.1.

Based on the gene annotation file of the reference genome Sscrofa11.1, we used Liftoff to annotate 21,472 protein-coding genes in MSjxau, with an average of 2.1 transcripts per gene. Additionally, we observed that regions with high gene density in the genome generally also exhibited higher GC content (Figure 2A). We also annotated repeat elements in the Meishan pig assembly. Overall, 42.06% of the regions in the Meishan pig assembly were marked as repetitive. Consistent with previous studies, LINEs were the most abundant repeat type, accounting for 19.40% of the Meishan pig assembly, followed by SINEs at 13.84%, LTRs at 4.72%, and DNA transposons at 2.33% (Figure 2B). We also annotated SD sequences in MSjxau, identifying a total of 56,257,287 SD sequences, of which 37.82% had repeat units greater than 10 kb. Furthermore, we compared the synteny between MSjxau and Sscrofa11.1 using dopplotly, revealing a good level of sequence consistency (Figure 2C), which supports the overall accuracy of the assembly. However, there were also 10,634,385 SNPs and 2,565,379 indels between the two genomes. Among these, 41,477 SNPs were predicted by SnpEff to be harmful mutations, and 4856 indels were predicted as frameshift variants. These variations may represent significant phenotypic differences between MS pigs and Duroc (DRC) pigs.

### 3.3. Comparison of Genomic Structural Variation 

**Figure 3:**
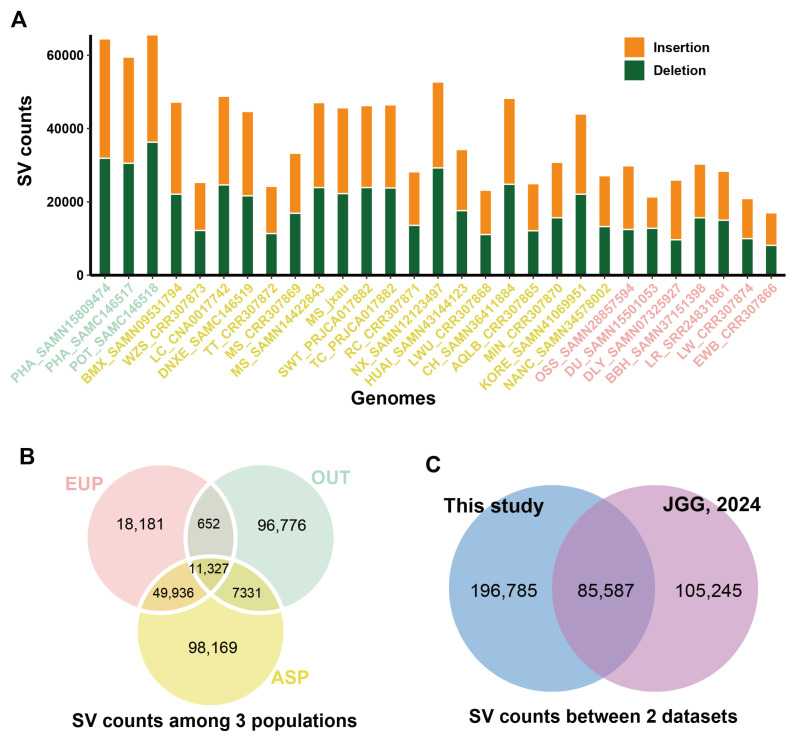
Statistical analysis of structural variations (SVs) in Meishan pigs. (**A**) The distribution of the number of structural variations detected in different pig breeds. (**B**) The statistical distribution of the number of structural variations in outgroup, Asian, and European populations. (**C**) Comparison and statistical analysis of the number of structural variations. PHA, *Phacochoerus africanus*; POT, *Potamochoerus porcus*; BMX, Bamaxiang pig; WZS, Wuzhishan pig; LC, Luchuan pig; DNXE, Diannan small-ear pig; TT, Tibetan pig; MS, Meishan pig; SWT, Shawutou pig; TC, Tunchang pig; RC, Rongchang pig; NX, Ningxiang pig; HUAI, Huai pig; LWH, Laiwu pig; CH, Chenghua pig; AQLB, Anqingliubai pig; MIN, Min pig; KORE, Korean minipig; NANC, Nanchukmacdon pig; OSS, Ossabaw minipig; DU, Duroc pig; DLY, Duroc × Landrace × Yorkshire pig; BBH, Babraham pig; LR, Landrace pig; LW, Largewhite pig; EWB, European wild pig; EUP, European pig; ASP, Asian pig; OUT, outgroup.

The MSjxau genome, along with 28 other publicly available genomes, was compared to the Sscrofa11.1 genome to detect genomic structural variations (SVs) (Table 2). Compared to Sscrofa11.1, an average of 37,440 SVs (18,658 insertions and 18,782 deletions) were identified for each genome. More SV loci were detected in the outbred pig genomes compared to the Asian and European pigs (Figure 3A). Subsequently, we merged the SVs from the 29 genomes to obtain a non-redundant set of SVs. A total of 282,372 SVs were identified, covering 78.75 Mb of the genome. We found that 11,327 SVs were shared across the Asian, European, and outbred pig genomes, while the majority of SVs were population-specific (Figure 3B). Finally, by comparing our results with recent studies on pig pan-genome SV genotyping, we identified more variation sites, with a total of 196,785 SVs. This provides additional valuable information for future SV analysis based on pan-genomes [61] (Figure 3C).

### 3.4. The Selective Domestication Between Chinese Wild Boar and Meishan Pig

**Figure 4:**
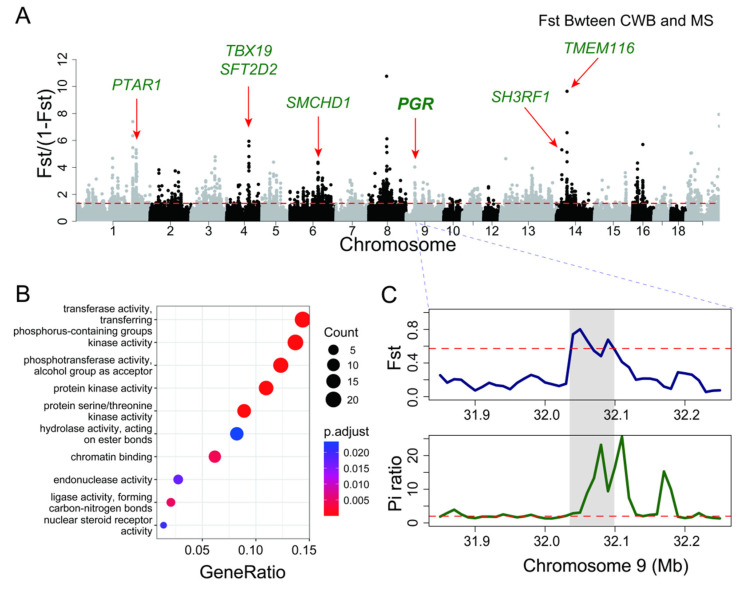
Selective regions on SVs between the CWB and the Meishan genome assembly. (**A**) Statistic Fst was plotted for selected SVs with threshold Fst value ≥ 0.01. (**B**) Pathway enrichment of a gene set. (**C**) Local display of Fst and nucleotide diversity ratio in chr9:31.9-32.2 Mb region. Note that the PGR gene overlaps with this selective sweep region.

We downloaded second-generation sequencing data for 44 Meishan pigs and 45 Chinese wild boars from public databases, and based on the Sscrofa11.1 reference genome, we identified a total of 7,671,328 genetic variants across 89 samples. Selective sweep analysis was performed using both FST (fixation index) and nucleotide diversity (π) methods [62], identifying 716 selective regions, which involved 408 protein-coding genes (Figure 4A). GO functional analysis revealed that these genes were significantly enriched in pathways related to chromatin binding, transferase activity, and protein kinase activity (Figure 4B). The candidate genes most strongly associated with Meishan pig domestication include *TMEM116, TBX19, SFT2D2, PTAR1, SMCHD1, PGR*, and *SH3RF1*. Among them, *TBX19*, a transcription factor involved in developmental process regulation, has been shown in several studies to be significantly associated with body size. The *PGR* gene, involved in multiple processes including the response to gonadotropin stimulation, plays a key role in significantly enhancing reproductive capacity, suggesting its potential link to the high reproductive traits of Meishan pigs (Figure 4C, Table 3).

**Figure 5:**
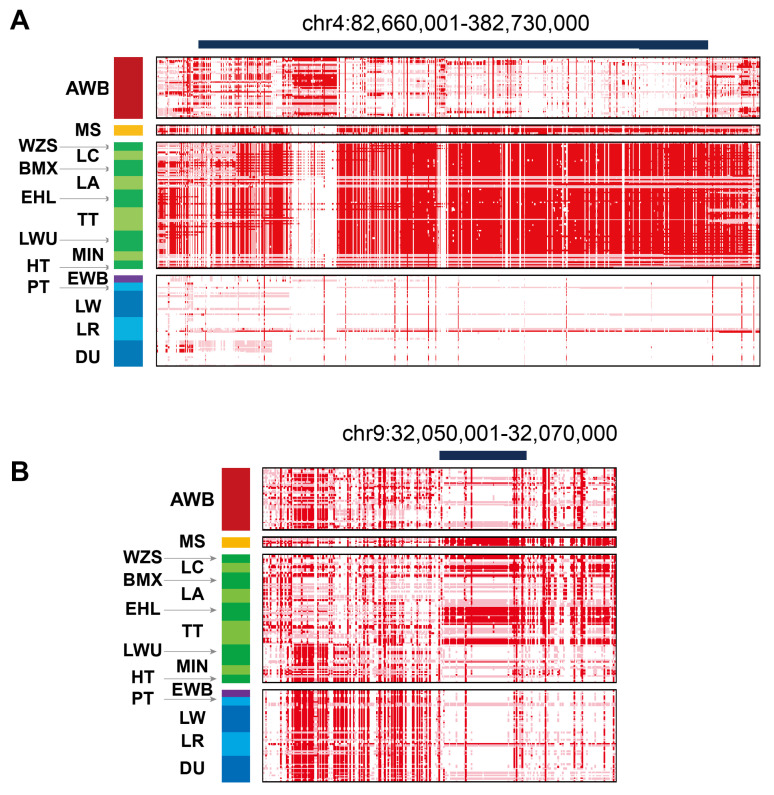
The degree of haplotype sharing in pairwise comparisons among 16 populations. (**A**) Haplotype sharing in interval chr4:82,660,001-82,730,000; (**B**) Haplotype sharing in interval chr9:32,050,001-32,070,000. AWB, Asian wild pig; MS, Meishan pig; WZS, Wuzhishan pig; LC, Luchuan pig; BMX, Bamaxiang pig; LA, Lean pig; EHL, Erhualian pig; TT, Tibetan pig; LWU, Laiwu pig; MIN, Min pig; HT, Heitao pig; EWB, European wild pig; PT, Pietrain pig; LW, Largewhite pig; LR, Landrace pig; DU, Duroc pig.

Next, we collected a total of 203 high-depth NGS datasets from 16 pig breeds as part of a previous project in our lab [5]. Our analysis revealed that *TBX19* and *PRG*-related selective haplotypes are specific to Asian pig breeds (Figure 5A,B). Notably, the *PRG* haplotypes are consistently present only in certain Asian breeds, such as the Erhualian pig. Interestingly, some individuals of the Landrace breed also exhibit genotypes that align with those found in the Meishan pig (Figure 5B).

## 4. Discussion

In this study, we constructed a chromosome level genome assembly for Meishan pigs with good accuracy, continuity, and completeness. A strong collinear relationship exists between MSjxau and the reference genome Sscrofa11.1, although structural variation and sequence differences due to different breeds are also observed. Currently, only 17 porcine chromosome level genomes have been assembled in the NCBI database using the PacBio or Nanopore sequencing data. Among these, A genome of the MS pig chromosome based on multiple sequencing techniques has also been reported recently [26]. The MSjxau we assembled is close to the quality of the genome previously assembled. The two MS genomes reflect the genetic diversity between different individuals of the same breed. Therefore, providing additional chromosome-level genome assemblies is essential for advancing domestic pig genomics research. Notably, one application of chromosomal-level genome is to construct a graphical pan-genome, which is conducive to fully and accurately mining structural variation. In this study, the MSjxau genome was integrated with 28 other genomes, and a total of 122,306 SVs were identified of which with 69.68% were new, which is significant for the analysis of using graph-based genome for genotyping of NGS data [61].

High-quality NGS data are the basis for accurate detection of population-level genetic variation. Here, we analyzed 45 CWB and 44 MS samples to precisely identify genetic variation between the two populations. Through selective sweep analysis, we identified 716 significantly differentiated regions containing numerous candidate genes under selection. Including *TBX19*, a gene that has been identified as being associated with the domestication history of Chinese native pigs in our previous study [63]. We also focused on the *PGR* gene, which encodes the progesterone receptor and plays key roles in reproductive tissues and thus impacts mammalian fertility [64]. Previous research has shown that progesterone receptor alleles derived from Neanderthals promote modern human fertility [65]. Therefore, we hypothesize that the *PGR* gene contributes to the high reproductive performance of the Meishan pig [66], which may have significant implications for the breeding and improvement of reproductive traits in domestic pigs. Additionally, the biological functions of selected genes are also involved in neurodevelopment, cell signaling, gene regulation, and lipid metabolism. For instance, *NRXN1* is closely linked to synaptic connectivity and neural function. mutations in this gene can lead to neurodevelopmental disorders, which may be associated with the docile behavior of domesticated pigs [67]. *SFT2D2* contributes to cell membrane stability and lipid metabolism [68], while *SMCHD1* is associated with chromosome stability and gene silencing, playing a key role in X chromosome inactivation [69]. Furthermore, *SH3RF1* affects cell growth by regulating cell signaling and protein degradation [70]. The gene referenced as *TMEM116* is also crucial for cell membrane stability and material transport [71]. The functions of all these genes can significantly impact various physiological processes, particularly in how cells adapt to environmental changes.

Due to issues such as inbreeding, the genetic diversity of Meishan pigs has decreased, leading to a narrow gene pool. This reduction in genetic diversity has negatively impacted their adaptability to environmental changes and resistance to diseases [72]. Furthermore, the rampant spread of African swine fever has caused some local Chinese pig breeds to approach extinction, and the Meishan pig is facing a similarly severe situation [73]. The rapid growth rate and high feed conversion efficiency of foreign superior pig breeds (such as Landrace and Duroc) have allowed these breeds to dominate the pig farming market, further limiting the breeding opportunities for local Chinese breeds like the Meishan pig. To address this, we recommend that breeders adopt genomic selection methods to identify and further propagate individuals with exceptional performance. Specifically, emphasis should be placed on incorporating favorable haplotypes, such as those associated with improved growth and reproductive traits, including the *PGR* and *TBX19* haplotypes identified in this study. Furthermore, government departments need to invest more in policies and funding to support the conservation of local breeds. At the same time, through policy guidance, local governments and farming enterprises should be encouraged to participate in breed conservation efforts. Efforts should also be made to promote the unique advantages of Meishan pigs, such as high reproductive capacity and strong adaptability, in order to secure a unique position in a broader market.

This study also has some limitations; for example, the MSjxau genome was scaffolded based on Sscrofa11.1 [1], without considering for large structural variations between breeds, which may have introduced a small number of structural errors. Additionally, we only achieved a chromosome-level assembly, leaving many gaps and assembly errors in highly complex regions. Several species have already completed T2T genome assemblies using HiFi sequencing technology [74,75], a standard we have not yet met. Therefore, future population-level T2T genome analysis will be an important research direction.

## 5. Conclusions

In conclusion, the Meishan pig genome assembly represents an important genetic resource for Chinese indigenous pigs. The high-quality chromosome-level genome enhances our ability to capture and characterize structural variations (SVs), particularly those carrying important genes. Importantly, selective sweep analysis between the Chinese wild boar and the Meishan pig revealed several key candidate genes, such as *TBX19* and *PGR*, linked to agronomic traits. Therefore, this study provides a foundation for improving breeding strategies and conservation of domestic pigs.

## Figures and Tables

**Table 1:** Statistics of the Meishan pig assembly and the reference genome assembly of the pig (Sscrofa11.1).

Assembly	MSjxau	Sscrofa11.1
Total length of assembly (Gb)	2.45	2.5
Min length (bp)	1008	15,096
Max length (Mb)	292.79	274.33
Number of scaffolds	1144	613
Number of gaps	466	544
Quality values (QV)	37.06	NA
Scaffold N50 (Mb)	139.17	138.97
Contig N50 (Mb)	15.62	48.23

**Table 2:** The basic classification of 28 public available pig genomes and MSjxau genome.

Assembly	Groups	Levels	Assembly	Groups	Levels
PHA_SAMN15809474	Outgroup	Chromosome	LC_CNA0017742	Asian	Contig
PHA_SAMC146517	Outgroup	Chromosome	TT_CRR307872	Asian	Contig
POT_SAMC146518	Outgroup	Chromosome	MS_CRR307869	Asian	Contig
BMX_SAMN09531794	Asian	Chromosome	RC_CRR307871	Asian	Contig
DNXE_SAMC146519	Asian	Chromosome	LWU_CRR307868	Asian	Contig
MS_SAMN14422843	Asian	Chromosome	AQLB_CRR307865	Asian	Contig
MS_jxau	Asian	Chromosome	MIN_CRR307870	Asian	Contig
SWT_PRJCA017882	Asian	Chromosome	OSS_SAMN28857594	European	Chromosome
TC_PRJCA017882	Asian	Chromosome	DU_SAMN15501053	European	Chromosome
NX_SAMN12123497	Asian	Chromosome	DLY_SAMN07325927	European	Chromosome
HUAI_SAMN43144123	Asian	Chromosome	BBH_SAMN37151398	European	Chromosome
CH_SAMN36411884	Asian	Chromosome	LR_SRR24831861	European	Contig
KORE_SAMN41069951	Asian	Chromosome	LW_CRR307874	European	Contig
NANC_SAMN34578002	Asian	Chromosome	LW_CRR307874	European	Contig
WZS_CRR307873	Asian	Contig			

**Table 3:** A fraction of domesticated genes in MS pigs based on Fst and Pi statistics.

Region	Gene	Top Fst	Description
chr1:223030001-223100000	*PTAR1*	0.841	Protein prenyltransferase activity
chr3:90190001-90220000	*NRXN1*	0.827	Nervous development
chr4:82660001-82730000	*TBX19*	0.848	Developmental processes
chr4:82660001-82730000	*SFT2D2*	0.856	lipid metabolism
chr6:103930001-103970000	*SMCHD1*	0.814	Epigenetic silencing
chr9:32050001-32070000	*PGR*	0.801	Progesterone Receptor
chr14:20450001-20470000	*SH3RF1*	0.841	Protein ligase activity
chr14:39710001-39780000	*TMEM116*	0.906	Transmembrane protein

## Data Availability

The assembly of the MSjxau genome can be obtained from https://github.com/jxlabWzZ/MS_jxau_genome_assembly.
